# Myricetin Apoptotic Effects on* T47D* Breast Cancer Cells is a *P53*-Independent Approach

**DOI:** 10.31557/APJCP.2020.21.12.3697

**Published:** 2020-12

**Authors:** Mitra Soleimani, Nayereh Sajedi

**Affiliations:** *Department of Anatomical Sciences, Isfahan University of Medical Sciences, Iran. *

**Keywords:** Myricetin, flavonoid, T47D, apoptosis

## Abstract

**Objective:**

Using nutraceuticals in cancer therapy is a strategy contributing with other approaches to promote apoptosis in cancer cells. Myricetin is a polyphenol flavonoid that forms main ingredients of various type of foods and beverages. The inducing properties of myricetin in apoptosis is reported by several investigations. The present study aimed to assess apoptotic effects of myricetin on *T47D* breast cancer cells and to evaluate part of the mechanisms of action.

**Materials and Methods:**

*T47D* breast cancer cells were assigned into five groups: control (cells in normal condition), myricetin (cells treated with myricetin IC50 concentration) in two different incubation times (24, 48 and 72 hours). MTT assay, annexin v assay, flow cytometry, caspase-3 assay and Real-time PCR were used to evaluate apoptosis in breast cancer cells.

**Results:**

The expression rate of apoptotic genes *caspase-3, caspase-8, caspase-9*, the ratio of *BAX /Bcl-2* as well as the expression of *P53, BRCA1, GADD45 *genes were increased significantly after treatment of* T47D *breast cancer cells with myricetin. Annexin v assay confirmed significant expression of annexin as were displyed by flow cytometry.

**Conclusion:**

Myricetin enhances apoptosis in *T47D* breast cancer cells by evoking both extrinsic and intrinsic apoptotic pathways. myricetin may practices its apoptotic properties on *T47D* cells through inducing *BRCA1- GADD45* pathway.

## Introduction

Breast cancer is the second causes of death and the most prevalent cancer among women in the world (Boulton, 2006). Due to unpleasant side-effects of chemotherapy and their influences on healthy tissues and organs, researchers are trying to find various natural ways to induce apoptosis in cancer cells (Wu et al., 2012). Polyphenols are one of the most abundant natural compounds that are found in plants and flavonoids (Martínez-Pérez, 2016). There are several reports on the role of these compounds in inhibiting the growth of cancer cells. Flavonoids are natural polyphenols constituents with the anticancer properties (Jiao and Dong Zhang, 2016; Ahn et al., 2018). Plants are highly rich in flavonoids as secondary plant metabolites (Weng and Yen, 2012 ). Studies have shown that flavonoids have various biological functions, such as changing epigenetics (Dashwood, 2007; Gilbert and Liu, 2010). They can increase the expression level of tumor suppressor *mRNAs *and induces apoptosis in the cell (Harkin et al., 1999; Gilbert and Liu, 2010). Myricetin is a flavonol which is found abundantly in nature (Li and Ding, 2012; Shin et al., 2013; Shiomi et al., 2013) with potent antioxidant activities (Erdman, 2006). Use of myricetin as a potent flavonoid in cancer therapy is an understudy approach in treatment of breast cancer. 

The role of mutations of tumor suppressor genes in onset and progression of many type of cancers is well recognized (Lee, 1989). Human breast cancer is associated with genetic and epigenetic alterations in a number of tumor suppressors (Perera and Bardeesy, 2012). Mutation in many tumor suppressor genes such as *TP53, BRCA1, *and *BRCA2* involve in the onset and progression of breast cancer (Rice et al., 2000; Dagdemir et al., 2013). 

*P53* gene is the most commonly mutated gene in several type of human cancers. It is mutated in approximately in 50% of all cancers. However, the overall frequency of *P53 *mutation in breast cancer is approximately 20% (Hollstein et al., 1991). 

Unlike other tumor suppressor genes that their mutation lead to their silence or downregulation, the majority alterations in *P53* leading to constitutive expression of mutant *P53* in cancer cells. This *P53 *mutations abolishes the function of wild- type P53 but based on the reports, it also aggravates the tumorigenesis. It is believed that mutations in the *P53* gene is leading to changes in the conformation of the p53 protein. This missense mutation lead to accumulation of p53 protein in cells. In other words, mutant *P53* contribute to tumorigenesis and mediates survival of the breast cancer cells ( Lim et al., 2009). In other words, mutatation of the* P53* gene, not only suppresses its tumor suppressor functions, but also increases the invasiveness in the cancer cells.

T47-D cell line is an epithelial type breast cancer which is derived for the first time from pleural effusion of an elderly patient. It is specialized with two characteristics: first it contains progesterone receptors that are not controlled by estradiol. Second, it is mutated for *P53 *but wild type* BRCA1* gene (Holliday and Speirs, 2011). The *BRCA1* gene (*BReast CAncer 1*) was the first gene was identified to create potentiality to breast cancer and its mutation was reported abundantly in familial breast cancers (Boulton, 2006; Lee and Muller, 2010). In sporadic breast cancer, its mutation is rare however in nearly 30% of cases it is downregulated (Yang et al., 2001). 

Apoptotic events are categorized into two pathways in which either mitochondria (internal pathway) or death receptors (external pathway) are involved. In the internal pathway, death signals lead to changes in mitochondrial membrane permeability; result in the release of the first set of proapoptotic factors such as cytochrome C. Cytochrome C binds to and activates Apaf-1 in the cytoplasm (Jiang et al., 2005). Subsequently, Caspase 9 is activated which in turn activates enzymes involved in cell death such as Caspase 3 and DNase (Nagata, 2000; Singh et al., 2007). External pathway is associated with Fas and TNF death receptors. These receptors induce Caspase 8 activity, which is followed by activation of downstream Caspases such as Caspase 3 (Park, 2005). 

*P53* tumor suppressor gene induces cell apoptosis through both internal and external signaling pathways. BRCA1 induces cell apoptosis in both TP53-dependent and TP53-independent pathways. In TP53-independent pathway, c-Jun N-terminal kinase (JNK) signaling pathway and the *GADD45* gene are involved (Fabbro et al., 2004; Ostrakhovitch and Cherian, 2005; Choene et al., 2012; Kim, 2017). 

This study set out to investigate the apoptotic effects of myricetin on *T47D* breast cancer cell line that is mutated for P53. Noticing the important roles of the *BCL2, BRCA1 *and *GADD45* genes stated earlier, the expression changes of these genes following treatment with myricetin in the *T47D* cells also aimed to be determined.

To assess the biological effects of myricetin on P53- mutated breast cancer cells, we used the T47D that is a P53- negative breast cancer cell line. We also aimed to investigate part of possible involved mechanism.

## Materials and Methods

*Cell culture*

T47-D breast cancer cells were purchased (National Cell Bank, Pasteur Institute of Iran, Tehran) and cultured in DMEM / F12 medium containing 10% FBS (Sigma, USA) and supplemented with 1% penicillin and streptomycin (Sigma, USA). Cells incubated for downstream experiments under standard conditions, 37°C and 95% humidity.

*MTT assay and determination of myricetin IC*_50_

To reach a 100 mmol/L stock solution of myricetin (Sigma, USA) compound dissolved in DMSO and stored at -20^o^C. The dosage of myricetin that inhibits half maximal proliferation of T47-D cells (IC_50_) (He et al, 2016), were determined as follows: 7,500 cells per well were cultured in 96-well plates and incubated overnight. Cells then were treated with a 200µl serial dilution of myricetin (175, 125, 100, 75, 50, 25 and10 μM) for 24 hours. Next, to determine the cell viability rate, cells got through MTT assay, using previously described protocol (Ahmadian et al., 2009). Assays included: medium-containing only wells, untreated control cells and test cells treated with myricetin in serial dilutions. MTT (2mg/ml) (Sigma, USA) diluted in DMEM-F12 and 20μL of it were added to each well. After incubation for 4 hours at 37°C, 95% humidity and 5% CO_2_, the medium was removed and to dissolve the resulted formazan, 200 μL DMSO were added to each well. The absorbance in each well then was measured at 570 nm wavelength using a microtiter plate reader. The wavelength of 650 nm was defined as the reference. The blanks were given values close to zero (+/- 0.1). IC_50 _dosage curve then were drawn. Based on the depicted curve, the concentration of 46μm was considered as myricetin IC_50 _dosage in this study.

*Experimental groups*

T47-D Cells were assigned into four groups: control (cells without treatment), myricetin group; cells treated with 46μm myricetin, that was divided itself into three groups regarding duration of treatment with myricetin (myr24h, myr48h and myr72h). A control group was designated for each time duration and accompanied with it to the end of experiment.

*Annexin V staining assay and flow cytometry*

During early apoptosis cell membrane asymmetry is rapidly lost without concomitant loss of membrane integrity. This results in the exposure of phosphatidylserine (PS) at the outer leaflet of the plasma membrane, which serves a physiological role in the recognition and subsequent removal of the dying cell by means of phagocytosis. Annexin V shows high affinity for PS residues in the presence of millimolar concentrations of Ca^2+^. These apoptotic cells can be distinguished from annexin V-negative living cells using flow cytometric procedure. By simultaneous probing of membrane integrity by staining with the nuclear dye propidium iodide (PI), apoptotic cells can be discriminated from necrotic cells (annexin V/ PI) (Schutte et al., 1998). Annexin V Apoptosis Detection Kit FITC (eBioscience, 88-8005, USA) were used for the assay. Following 24, 48 and 72 hours of treatment with 46µM myricetin, the cells were trypsinized and washed with PBS. After adding the binding buffer, 5 μl of Annexin V-FITC was treated. Cells were incubated at room temperature for 15 minutes and then washed with washing buffer. Finally, 200 µl buffer and 5 µl PI (Propidium Iodide) were added to the cells and apoptotic cells were counted by flow cytometry (Becton Dickinson, Heidelberg, Germany). The experiments were performed in triplicate independently at three different times. 

*Real-Time PCR*

The expression level of relevant genes was determined by Real-Time PCR. The cells were treated with 46µM myricetin for 24, 48, and 72 hours. Total RNA in all groups was extracted using the YTA Total RNA Purification Mini kit (Yekta Tajhiz Azma, Iran) according to the manufacturer protocol. After treatment with DNase I to remove genomic DNA, cDNA was reverse transcript using RevertAid ™ First Strand cDNA Synthesis Kit (Fermentas). Maxima SYBR Green ROX qPCR Master Mix kit (Fermentas) used according to the manufacturer’s protocol in an ABI StepOnePlus™ Real-Time PCR System (Applied Biosystems). The cycling parameters were as follows: 10 min at 95°C for initial denaturation, followed by 40 cycles of denaturation step at 95°C for 15s and annealing/ extension for 1 min at 60°C. β-actin was used as a reference gene for internal control. Data were analyzed by Comparative Ct (ΔΔct) method. These experiments were carried out in triplicate and were independently repeated at least 3 times. Gene-specific primer sequences for *P53, BRCA1, GADD45, BAX*, *Caspase 3, Caspase 8, Caspase 9,* and *Bcl-2* are presented in Table 1. 

*Caspase 3 activity assay*

To evaluate the activity of caspase-3, Fluorometric Assay Kit for *Caspase-3/ CPP32* (BioVision, Catalog, and K105-25) was utilized. *Caspase-3* is a critical executioner of apoptosis and responsible for the proteolytic cleavage of many key proteins. This assay is a fluorescent assay which detects the activity of caspase-3 in cell lysates. It contains a fluorogenic substrate (N-Acetyl-Asp-Glu-Val-Asp-7-amino-4-methylcoumarin or Ac-DEVD-AMC or -AFC) for* caspase-3*. During the assay, activated caspase-3 cleaves this substrate between DEVD and AMC, or AFC, generating highly fluorescent that can be detected using a fluorescence reader with excitation at 380 nm and emission between 420 - 460 nm. The more apoptotic cells in the sample, the more Caspase-3 activity and the more fluorescence emission generated (Ponder and Boise, 2019). The T47-D cells were treated with 46 µM myricetin for 24h, 48h, and 72h. Next, cells were trypsinized and washed with PBS. The pellete of cells suspend in 50 µl of chilled Cell Lysis Buffer. Then 50 µl of 2X Reaction Buffer (containing 10 mM DTT) added to each sample. Next, cells were incubated on ice for 10 minutes. Subsequently, 50 μL of 2X reaction buffer, 1 μL of DTT (1 M), and 5 μL of DEVD-AFC (1 mM) were added to the cell lysates. The reactions were incubated for 2 h at 37°C, 5% CO_2_, and 95% humidity. Finally, 50 μL of cell lysates were transferred in to a 96 well plate and the absorbance was read using a fluorometric spectrophotometer with 400 excitation and 505-nm emission filters. DEVD-AFC emits blue light at 400nm.

*Statistical analysis*

One-way analysis of variance (ANOVA) with Tukey post hoc test was performed using SPSS software package 25.0. to determine statistical significance among different groups. The quantitative data were presented as the mean ± standard deviation (SD). P-value <0.01 was considered as statistically significant level. 

## Results

*MTT assay and IC50 dose response curve*

**Figure 1:**
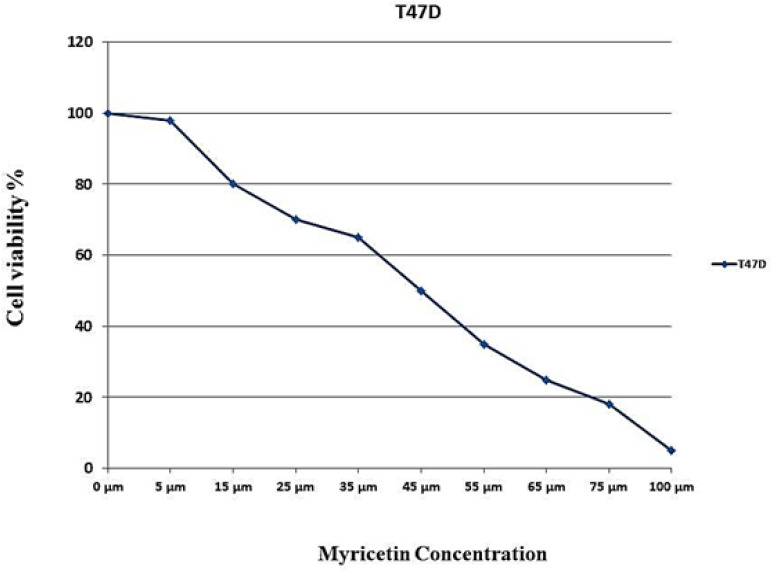
IC_50_ Assay for Half-Maximal Inhibitory Concentration Analysis of Myricetin in T47-D Cancer Cells after 24 Hours of Treatment. Cells were treated with or without the myricetin in serial dilutions (175, 125, 100, 75, 50, 25 and10 μM), and the relative amount of viable cells were estimated by measuring the absorbance of the cell suspension after incubation. MTT was carried out and a dose response graph of viability versus myricetin concentration used to calculate IC_50_ values for T47-D cells. The graph pointed the concentration of 46 µM as IC_50_ for myricetin

Approaching the results of MTT assay, a dose response curve for myricetin was depicted and 46 μM considered as IC_50_ (Figure 1).

*Cell viability assay*

**Figure 2:**
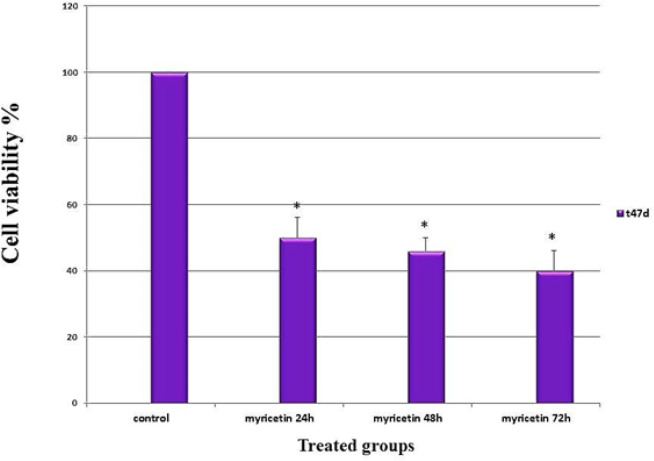
The Viability Assay of the T47-D Breast Cancer Cells Following Treatment with Myricetin as Evaluated by MTT Assay. Cells treated with half maximal inhibition concentration of myricetin (46 µM) for 24, 48 and 72 hours. The viability of the cells decreased significantly with time. Data is represented as mean ± SD. * p< 0.01

The results displayed that the viability of the cells decreased significantly following exposure to myricetin (p < 0.01). The anti-proliferative effects increased incrementally by time (Figure 2).

*Flow cytometry and Annexin v assay*

**Figure 3:**
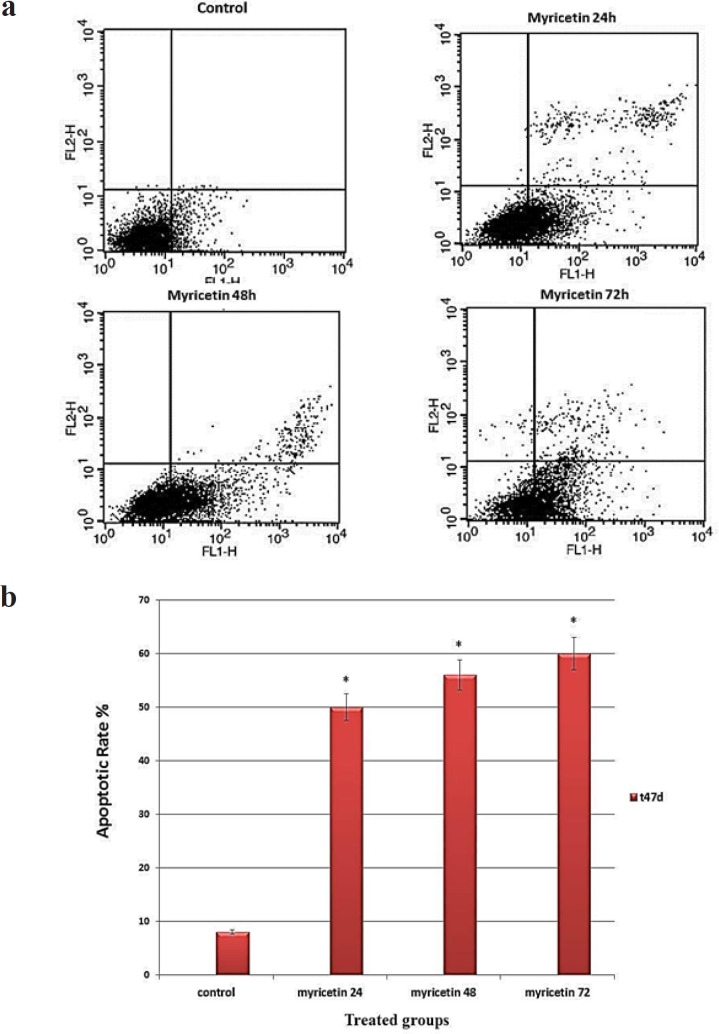
Flow Cytometry Analysis of Myricetin-Induced T47-D Cells. (A) The dot plot diagrams represent typical apoptotic and necrotic cell populations detected by Annexin V-FITC and PI staining. The lower left quadrants of the panels show viable intact cells, which were negative for Annexin V-FITC binding and excluded PI staining (FITC-/PI-); the upper right quadrants show late apoptotic cells, which were positive for Annexin V-FITC binding and PI uptake (FITC+/PI+). The lower right quadrants represent early apoptotic cells, positive for Annexin V-FITC and negative for PI (FITC+/PI-). (B) Bar graph shows average percentage of apoptotic cells. The values are presented as means ± SD. *p<0.01

Flow cytometry was performed to determine the rate of annexin v positive cells. The results showed a significant increase in apoptosis rate in T47-D cells treated with 46 μM myricetin IC_50_ concentration, (p < 0.01) (Figure 3). 

*Real-time PCR*

**Figure 4:**
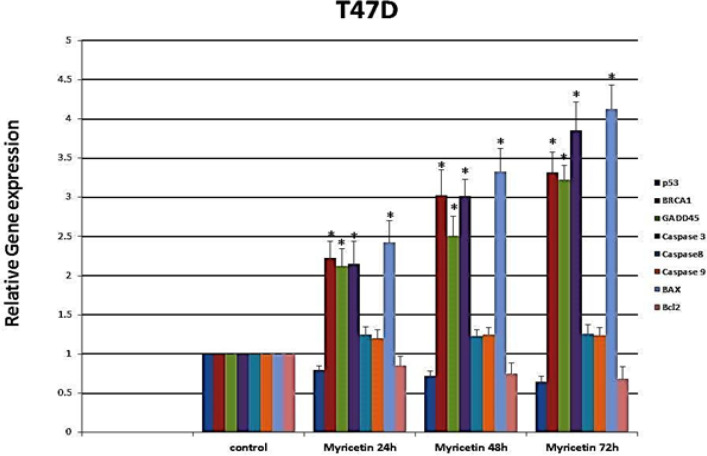
Real-Time PCR Gene Expression Analysis of T47-D Cells Treated by 46 µm Myricetin for 24, 48, and 72 hrs. The expression of BRCA1, GADD45, Caspase-3, and BAX increased. However, the expression level of P53, caspase 8, caspase 9; and Bcl-2 did not show a noticeable change compared to control groups. Data are represented as mean ± SD. *p<0.01

The expression level of *P53, BRCA1, GADD45, BAX, Caspase-3, Caspase-8, Caspase-9,* and* Bcl-2* was determined by Real-time PCR. The expression level of *BRCA1, GADD45, Caspase-3* and *BAX* genes increased significantly after treatment with myricetin. However, the expression level of *P53, caspase 8, caspase 9*; and *Bcl-2 *did not show a noticeable change compared to control groups (Figure 4).

*Caspase-3 activity assay*

**Figure 5:**
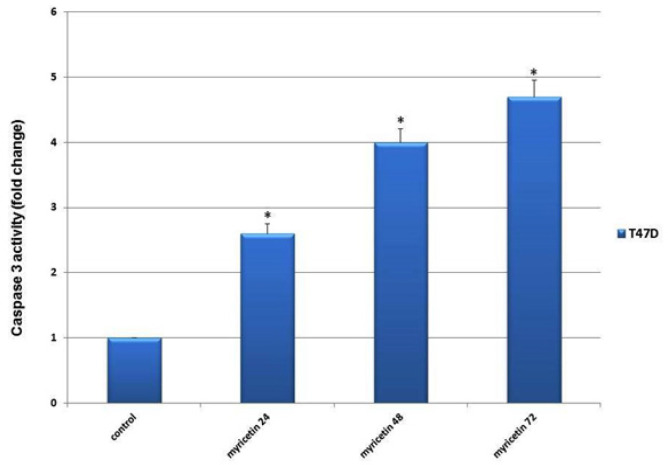
The Caspase-3 Activity in T47-D Breast Cancer Cells Plotted Against duration of Exposing Cells to 46 µm Myricetin (24, 48 and 72 hrs). The Caspase-3 activity increased with time significantly compared to control group. The data is expressed as the percentage of cells that show a cytoplasmic fluorescence intensity greater than 450 (Z’= 0.51)

The results showed a significant increase in Caspase-3 activity in the treated groups compared to the control groups. There also were shown that the pick of the caspase-3 activity was up to 72 hours after treatment with myricetin (Figure 5).

**Table 1:** Primers Used in Real-Time PCR

Primers ID	Primers Sequences
Bcl-2 Forward	AAAATACAACATCACAGAGGAAG
Bcl-2 Reverse	CTTGATTCTGGTGTTTCCC
Caspase 9 Forward	CCTTTGTTCATCTCCTGCTTAG
Caspase 9 Reverse	CCTCAAACTCTCAAGAGCACC
BRCA1 Forward	TGTTACAAATCACCCCTCAAG
BRCA1 Reverse	CCTGATACTTTTCTGGATGCC
GADD45 Forward	TTTTGCTGCGAGAACGAC
GADD45 Reverse	GAACCCATTGATCCATGTAG
Caspase 3 Forward	AGCACTGGAATGACATCTCG
Caspase 3 Reverse	ACATCACGCATCAATTCCAC
TP53 Forward	CACTCCAGCCACCTGAAGTC
TP53 Reverse	GCAAGCAAGGGTTCAAAGAC
BAX Forward	GGAGCTGCAGAGGATGATTG
BAX Reverse	GTCCAATGTCCAGCCCATG
Caspase 8 Forward	ACTGGATGATGACATGAACCTG
Caspase 8 Reverse	GCTGAATTCTTCATAGTCGTTG
Beta actin Forward	TTCGAGCAAGAGATGGCCA
Beta actin Reverse	CACAGGACTCCATGCCCAG

## Discussion

The present study aimed to assess the effect of myricetin, one herbal potent antioxidant, on the apoptosis of T47-D breast cancer cells. Numerous studies have shown that myricetin exert influential effects on malignancies such as breast, colorectal, bladder, lung, skin, and esophageal cancers (Baur and Sinclair, 2006; Zhanget al., 2013; Phillips et al., 2011; Zang et al., 2014; Kim et al., 2014). However, the precise molecular mechanism of myricetin in induction of apoptosis in cancer cells has to be clarified.

It is believed that phytoestrogens, including flavonoids, conducive the reduction in the expression of mutated *P53*, but promote the expression of the wild-type *P53*. Phytosterogenes act as the ligands for the ERα+ and have different effects. They may be estrogenic or anti-estrogenic. It is reported that in cell lines with mutated P53, flavonoids function as antiestrogenic and reduce the expression of *P53* by reducing the expression of *ERα*. Mutated P53 play oncogenic rules and has aggravates in proliferation and prohibition in cnacer cells (Avila et al ., 1994; Takagaki et al., 2005; Saluzzo et al., 2016). 

It has been proved that Quercetin, a flavonoid that is a common component of the human diet, mediates the down-regulation of mutant *P53* gene in the human breast cancer cell line MDA-MB468. Quercetin did not affect the mRNA levels of P53, but prevented the accumulation of newly synthesized p53 protein (Avila et al., 1994). In our study, considering the steady-state low level gene expression of *P53* before and after treatment with myricetin, it is interpreted that the apoptotic effects of myricetin on T47-D cells is being exerted by a mechanism independent of wild-type *P53* expression. However, we do not suggest the state of mutant *P53* after treatment with myricetin.

According to studies conducted on the effect of myricetin on various cancer cell lines, the genes involved in apoptosis and their expression level highly depend on the type of cell line (Avila et al., 1994; Jiao and Dong Zhang, 2016; Kim, 2017; Zheng et al., 2017). To assess the biological effects of myricetin on P53- mutated breast cancer cells, we used the T47-D that is a P53- negative breast cancer cell line. Studies on the T47-D cell line are limited and the effect of myricetin on the expression of *BRCA1* tumor suppressor gene and *GADD45* has not been investigated so far. 

*P53* is the most commonly mutated gene in several types of human cancers. It is mutated approximately in 50% of all cancers. However, the overall frequency of *p53* mutation in breast cancer is approximately 20% (Hollstein et al., 1991). Weiss et al., (2010) reported that the deletion of *P53* gene in human breast epithelial cells led to the chromosal disability. It is reported that mutant p53 proteins gain carcinogenic properties that promote cancer cell invasiveness and metastasis (Tanaka et al., 2018). 

In wild-type P53 breast cancer cell lines, it is suggested that flavonols induce apoptosis by regulating *P53* gene expression. For instance, kaempferol induces apoptosis by up-regulation and phosphorylation of P53 ( Choi and Ahn, 2008). 

According to the studies on liver cancer HepG2 and leukemia HL-60 cell lines, myricetin-induced apoptosis were reported to be due to induction of *P53* expression and increased activities of Caspase 3 and 9 (Wang et al., 1999; Zhang et al., 2010; Zhang et al., 2013). 

*BRCA1* is a tumor suppressor gene that its expression is usually reduced or absent in approximately 30% of sporadic cases (Esteller et al., 2000; Yang et al., 2001). *BRCA1* tumor suppressor gene induces apoptosis through both intrinsic and extrinsic pathways. However, *BRCA1* acts through both P53-dependent and P53-independent pathways and JNK signaling pathway and GADD45 are involved in P53-independent pathway (Fabbro et al., 2004; Ostrakhovitch and Cherian, 2005; Choene et al., 2012; Kim, 2017). The result of the present study showed that the expression of *BRCA1* and *GADD45* increased following treatment with myricetin.

The precise molecular mechanism of myricetin has not been elucidated. In the previous studies, the apoptosis induced by myricetin has been declared to be Caspase-dependent or Caspase-independent. 

In the intrinsic pathway of apoptosis, increased ratio of BAX/Bcl-2 and activation of Caspase 9 are responsible in cell apoptosis. In the extrinsic pathway, activation of Caspase 8 and 10 initiate apoptosis (Nagata, 2000; Jiang et al., 2005; Singh et al., 2007). 

The results of this study presented that the rate of cell apoptosis increased following the treatment of T47-D breast cancer cells with myricetin. 

According to a report, myricetin induced apoptosis in human promyeloleukemic cell line HL-60 by increasing *Caspase 3* and *Caspase 9* activities and B*AX/Bcl2 *expression (Wang et al., 1999). Another study on MCF-7 cell lines revealed that various concentrations of myricetin induce apoptosis in breast cancer cell lines. In addition, myricetin activated the expression of *Bax* protein and increased the activity of Caspase 3 (Jiao and Dong Zhang, 2016). 

The promotion of the expression of *caspase 3*, and *BAX/Bcl-2* ratio elevated with time, while the expression of *caspase 8* and *caspase 9* remained unchanged during the experiment. 

Finally, considering the presence of wild type *BRCA1 *tumor suppressor gene in *T47-D* cell line and the incremental effects of myricetin on the expression of *BRCA1, BAX, GADD45 *and *Caspase 3* genes, myricetin may exert its influencies in p53- mutated breast cancer cells by regulating *GADD45 *and *BRC1*. In conclusion, using myricetin could be suggested as a complementary approach in treatment of breast cancer with *P53* mutations. 
